# Wilson's disease presenting with arthralgia: a case report

**DOI:** 10.3389/fped.2026.1781752

**Published:** 2026-04-10

**Authors:** Yiyuan Li, Yang Wen

**Affiliations:** Key Laboratory of Women and Children Diseases, Laboratory of Birth Defects and Related Diseases of Women and Children (Sichuan University), Ministry of Education, Department of Pediatrics, West China Second University Hospital, Sichuan University, Chengdu, China

**Keywords:** arthralgia, child, liver dysfuction, pediatric, wilson's disease

## Abstract

**Backgrounds:**

Wilson's disease (WD) is a rare autosomal recessive disorder of copper metabolism characterized by impaired hepatic copper excretion and progressive multisystem copper accumulation. While hepatic and neuropsychiatric manifestations predominate, arthralgia as an initial presentation is uncommon. We report a child with WD who presented initially with joint pain.

**Case presentation:**

A ten-year-old patient experienced recurrent joint pain, predominantly in the lower limbs, and physical examination revealed tenderness in the right ankle joint and hepatomegaly. Laboratory investigations demonstrated liver dysfunction, including elevated alanine aminotransferase, aspartate aminotransferase, and total bilirubin levels. Further evaluation confirmed the diagnosis of WD based on the presence of Kayser–Fleischer rings on ophthalmologic examination, decreased serum ceruloplasmin levels, increased 24-hour urinary copper excretion, and identification of a homozygous mutation in the ATP7B gene. Treatment with penicillamine and zinc supplementation was initiated, after which the child experienced gradual improvement in joint symptoms along with normalization of liver biochemical parameters.

**Conclusion:**

WD should be considered in the differential diagnosis of children presenting with unexplained arthralgia, particularly when accompanied by abnormal liver biochemical findings.

## Introduction

Wilson's disease (WD) is an autosomal recessive disorder caused by mutations in the ATP7B gene, leading to impaired hepatic copper transport and subsequent copper deposition in various tissues. The initial clinical presentation is commonly characterized by hepatic, neurologic, or psychiatric/behavioral disturbances (1). In addition, there can be involvement of the eyes, kidneys, heart, bones and joints, blood, endocrine system, and sleep patterns (2). Arthritis as the initial manifestation of WD, however, is relatively uncommon and often underrecognized. Here, we report a case of WD in a child presenting primarily with osteoarticular symptoms, with the aim of increasing clinical awareness and improving early recognition of this atypical presentation of WD.

## Case presentation

A 10-year-old boy was admitted to the hospital with a five-month history of recurrent pain in the lower limb joints. The symptoms initially manifested as pain in the right knee joint without an identifiable trigger. The pain was mild in intensity and typically resolved spontaneously within approximately one minute. Mild swelling of the affected joint was also observed. Over time, the pain gradually involved the left knee and the right ankle joints. In addition, the patient developed mild difficulty in walking, suggesting some degree of gait instability. The child had no significant past medical history and no relevant family history.

The patient exhibited generalized abdominal tenderness on physical examination. The liver was palpable with a firm consistency, extending approximately three centimeters below the right costal margin along the midclavicular line, with a liver span of twelve centimeters on percussion. The spleen was not palpable in the midclavicular line. Localized swelling of the right ankle joint was observed, accompanied by mildly increased skin temperature and tenderness. The remainder of the physical examination was unremarkable.

Laboratory investigations revealed abnormal liver function parameters, including alanine aminotransferase (ALT) 59 U/L (reference range <49 U/L), aspartate aminotransferase (AST) 110 U/L (reference range <40 U/L), total bile acids (TBA) 43.5 µmol/L (reference range 0–10 µmol/L), total bilirubin 33.0 µmol/L (reference range 5–23 µmol/L), direct bilirubin 12.3 µmol/L (reference range <8 µmol/L), and indirect bilirubin 20.7 µmol/L (reference range <20.7 µmol/L). Serum albumin, coagulation profile, and blood ammonia levels were within normal limits. Serum ceruloplasmin was markedly decreased at 0.042 g/L (reference range 190–670 mg/L), and serum copper concentration was reduced to 346.7 µg/L (reference range 800–1,900µg/L). Twenty-four-hour urinary copper excretion was significantly elevated at 166.4 µg/24 h (reference range 15–30 µg/24 h). Autoantibody testing revealed positive antinuclear antibodies with a speckled pattern at a titer of 1:100. Other laboratory parameters were within normal limits, including complete blood count, C-reactive protein, procalcitonin, erythrocyte sedimentation rate, urinalysis, lipid profile, fasting blood glucose, renal function, electrolytes, rheumatoid factor, serological and cellular immunological tests, human leukocyte antigen B27 typing, and the autoimmune liver disease antibody panel. Additional testing for antibodies against hepatitis A and E viruses, interferon-gamma release assay, and bone marrow examination were unremarkable.

Contrast-enhanced magnetic resonance imaging (MRI) of the upper abdomen revealed abnormal liver morphology and signal intensity, splenomegaly, and mild thickening of the portal vein, findings suggestive of liver cirrhosis with possible regenerative nodules. Contrast-enhanced MRI of the sacroiliac joints demonstrated normal bilateral appearance. Mild soft tissue swelling and a small joint effusion were noted in the right ankle. Additionally, abnormal signal intensity was observed in the distal metaphysis and epiphysis of the right tibia and fibula, as well as in the navicular bone of the right foot. Chest computed tomography and contrast-enhanced brain MRI were unremarkable. Ophthalmologic examination revealed yellow-brown pigment deposition at the corneal margins in both eyes, consistent with Kayser–Fleischer rings.

Genetic analysis identified a homozygous missense variant, c.2975C > T (p.Pro992Leu), in the ATP7B gene. Both parents were found to be heterozygous carriers of this variant.

According to the Leipzig scoring system, the patient achieved a total score of 10: Kayser–Fleischer rings observed on ophthalmic examination (2 points); serum ceruloplasmin <0.1 g/L (2 points); 24-hour urinary copper excretion exceeding twice the upper limit of normal (2 points); and a homozygous pathogenic mutation (4 points). These findings collectively supported the diagnosis of WD.

The patient was managed with a low-copper diet and pharmacologic therapy including penicillamine and zinc preparations. After one week of treatment, joint pain was alleviated, although mild tenderness persisted in the right ankle. At discharge, the patient continued oral penicillamine and zinc therapy. At one-month follow-up, the patient reported complete resolution of joint pain and tenderness. Liver function tests demonstrated improvement, with ALT 64 U/L, AST 78 U/L, total bile acids 21.6 µmol/L, and normal total bilirubin and coagulation profile. The clinical course is summarized in Table 1. The patient was prescribed longtime copper-chelating therapy and remains under regular outpatient follow-up.

**Table 1:** Timeline of clinical manifestations, laboratory findings, and treatment in the patient with WD.

Time point	Symptoms and signs	Laboratory findings and treatment
5 months prior to admission	Pain in the right knee joint	No examination or treatment performed
At admission	Pain in both knees and the right ankle joint	No examination or treatment performed
1 week later admission	Pain in both knees and the right ankle joint	Elevated liver enzymes, decreased serum ceruloplasmin, elevated 24-hour urinary copper excretion, positive Kayser–Fleischer rings; initiated low-copper diet, zinc acetate, and penicillamine
2 weeks later admission	Joint pain improved, residual tenderness	Continued low-copper diet, zinc acetate, and penicillamine after discharge
4 weeks later admission	Complete resolution of joint symptoms	Genetic testing revealed homozygous mutation in *ATP7B*; improved liver function

WD, Wilson's disease.

## Discussion and conclusions

WD is a rare inherited disorder of copper metabolism characterized by defective copper transport and accumulation in multiple organs. Hepatic manifestations are the most common initial presentation (3–5), including hepatomegaly, isolated splenomegaly, elevated serum aminotransferase levels, jaundice, acute hepatitis, cirrhosis, and even acute liver failure (4, 5). Neurological involvement represents the principal extrahepatic manifestation of copper toxicity, typically presenting with tremor, dystonia, cognitive and attentional impairment, as well as gait instability and incoordination. In addition, a variety of less common manifestations may occur, affecting the kidneys, eyes, heart, bones, and other organs (2).

The marked heterogeneity of clinical manifestations in WD often complicates its differentiation from other disorders. A major challenge lies in the early recognition of atypical or uncommon presentations of WD (6). Previous reports have indicated that the average time to diagnosis remains prolonged, often exceeding two years from the onset of symptoms (3, 7, 8). Articular manifestations have been reported as atypical and occasionally initial presentations in patients with WD, which may lead to misdiagnosis and subsequent adverse outcomes (9–12). For instance, one report described a young male patient with WD who initially presented to hospital because of a forearm fracture (12). In another case, a 30-year-old patient experienced progressively worsening pain involving both small and large joints for fourteen years before a diagnosis of WD was established (9). Additionally, a seven-year-old girl presented with lower limb pain and deformity and subsequently underwent corrective surgery, followed by a leg fracture; WD was diagnosed four years later when neurological symptoms eventually developed (10). Therefore, it is essential to consider and differentiate WD in patients presenting with arthritis or unexplained articular symptoms. Delayed diagnosis, inadequate treatment, or inconsistent management of WD may lead to irreversible neurological damage and, in severe cases, death.

The diagnosis of WD requires a comprehensive evaluation integrating biochemical, clinical, radiological, and genetic findings. Initial screening primarily focuses on abnormalities in copper metabolism, including serum ceruloplasmin levels and baseline 24-hour urinary copper excretion. Subsequently, assessment of characteristic extrahepatic manifestations—such as Kayser–Fleischer rings, neurological symptoms, and Coombs-negative hemolytic anemia—is essential. Cranial MRI is also recommended, particularly in children older than 10 years of age. If WD cannot be definitively confirmed or excluded through these evaluations, hepatic copper quantification in liver tissue (measured on a dry-weight basis) should be performed. Genetic testing of the ATP7B gene can establish a definitive diagnosis and is valuable for family screening (13). According to the Leipzig scoring system, a score ≥4 confirms the diagnosis of WD, a score of 3 indicates probable WD, and a score ≤2 makes the diagnosis unlikely (14). When the clinical and laboratory findings sufficiently support the diagnosis, pharmacological treatment should be initiated promptly. In the present case, the patient initially presented with lower limb joint pain. Subsequent evaluation revealed hepatomegaly and abnormal liver function. Further screening—including measurements of ceruloplasmin, serum copper, and urinary copper, ophthalmologic examination for Kayser–Fleischer rings, and genetic testing—ultimately confirmed the diagnosis of WD. Early and lifelong treatment represents the cornerstone of WD management. Following the implementation of a low-copper diet, treatment with copper-chelating agents such as penicillamine, and zinc salt therapy, the patient's joint pain improved and liver function partially recovered. Early diagnosis and timely treatment likely prevented the development of joint deformities or pathological fractures.

WD should be considered in the differential diagnosis of patients presenting with arthritis, particularly when accompanied by abnormal liver function tests. Prompt recognition and appropriate treatment can lead to favorable clinical outcomes and may prevent long-term complications resulting from copper accumulation in tissues.

## Data Availability

The original contributions presented in the study are included in the article/supplementary material, further inquiries can be directed to the corresponding author.
